# Promoting Health through Accessible Public Playgrounds

**DOI:** 10.3390/children10081308

**Published:** 2023-07-29

**Authors:** Mehrnoosh Movahed, Luca Martial, Tiiu Poldma, Monica Slanik, Keiko Shikako

**Affiliations:** 1 School of Physical and Occupational Therapy, McGill University, Montreal, QC H3G 1Y5, Canada; mehrnoosh.movahed@mcgill.ca (M.M.);; 2 School of Design, University of Montreal, Montreal, QC H3C 3J7, Canada

**Keywords:** children with disabilities, inclusive playground, accessibility, inclusion, policy, universal design

## Abstract

Every child, irrespective of socioeconomic status, ethnicity, or ability, deserves the fundamental right to experience play, which is a powerful and enriching activity that fosters their physical and mental health. Regrettably, most public play spaces hinder the complete inclusion of children with disabilities, with the main reason being a lack of universal accessibility. This study identified existing international and Canadian policies and community best practices related to inclusive playgrounds, and gathered stakeholders’ opinions on the present condition of playgrounds, including challenges faced by children with disabilities and recommendations to make playgrounds inclusive. The questionnaires were completed by 97 participants. In addition, 10 individual interviews and three focus groups were performed. Ten policy documents and five community best practices were found. Our investigation identified factors that influence the design and implementation of inclusive playgrounds, categorized into three main areas: physical, social, and political environments. The results indicate that children with disabilities lack opportunities to engage fully in the physical and social aspects of playgrounds and spontaneous play activities with their peers. Notably, children with multiple disabilities may not feel welcomed or included in existing public play spaces.

## 1. Introduction

Every day, around 800,000 children with disabilities in Canada face challenges to access their right to do what every child should: play [1,2]. This is happening in public spaces including playgrounds, sports venues, community centers that should be accessible, utilized, explored, and embraced by everyone. Play contributes to physical, intellectual, emotional, and social development of all children [3]. Playgrounds are not only an important place for physical activities but also space for children to meet and interact with other children, develop communication skills and social abilities, and develop a sense of community and ownership of their neighborhood and public areas [4,5]. Conversely, playgrounds can inadvertently contribute to exclusion and discrimination, and further extend existing inequities. By imposing limitations on the accessibility of public playgrounds, these spaces may induce feelings of inadequacy and failure among marginalized communities who are excluded from being able to fully participate. Therefore, public playgrounds hold equal significance for children with disabilities as they do for other children.

Play is a human right; the United Nations Convention on the Rights of the Children (UN CRC) states that “accessible and inclusive environments and facilities must be made available to children with disabilities, to enable them to enjoy their rights (Article 31) [6]. According to the Convention on the Rights of Persons with Disabilities (UN CRPD), State Parties must “ensure that children with disabilities have equal access with other children to participation in play, recreation, leisure and sporting activities, including those activities in the school system” (Article 30) [7]. However, according to a Canadian national survey, 69.7% of children with disabilities in Canada between 0 and 4 years old reported experiencing mild to moderate disadvantages while playing, while 8.8% reported severe disadvantages during play. For children aged 5–14 years, 44.3% faced challenges in transportation or leisure, highlighting the various environmental barriers that they encounter [2].

Children with disabilities are at a higher risk of exclusion in several forms of social participation, including leisure activities and free use of public spaces, which constitutes a violation of their human rights. Most current public play spaces preclude the full participation of children due to physical barriers, e.g., sand or woodchip ground surface, narrow pathways, and lack of ramp to play structures, as well as social barriers such as attitude of other children and adults toward children with disabilities. In addition, the fact that children with disabilities cannot play independently and are accompanied by adults may disrupt the typical peer-to-peer interaction and limit their opportunities for unrestricted engagement with other kids [8,9,10,11]. The aims of this study included (1) to identify the existing policy-related original articles, acts, legislations, and guidelines related to inclusive playgrounds, and (2) to gather stakeholders’ perspectives on the present condition of playgrounds, including challenges faced by children with disabilities and recommendations to make playgrounds inclusive.

## 2. Methods

We conducted an iterative mixed-methods design [12] to triangulate the environmental scan results of policies, quantitative surveys, and qualitative data collected through interviews and focus groups. 

### 2.1. Environmental Scan

To understand the context of regulating public play spaces such as playgrounds, and the existing local practices for inclusive playgrounds, we conducted an environmental scan of policy documents and community-based reports and documents describing best practices. 

#### 2.1.1. Policy Documents

The Canadian government comprises federal, provincial, and territorial and municipal governments, which share responsibilities and functions. The federal government sets forth priorities, national strategies, standards, and policy guidelines to be implemented by the provincial, territorial and municipal jurisdictions. We initially searched for the keywords on Google and government websites (federal, and then provincial and territorial). We also searched for secondary references within summaries of policies and references. We collected a variety of documents included: policy guidelines, legislation, acts, action plans, standards, and policy briefs regarding accessible or inclusive play spaces. The three main categories of the keywords were play, children with disabilities, and policy; the detailed keywords were playground, play space, public, school playground, inclusive/adapted, accessible, accessibility, usable, usability, universal design, children with disabilities, community development, social capital, policy, guideline, legislation, convention, act, action plan, standard, and framework. All different types of functional limitations (i.e., physical, intellectual, communication, and sensory impairments) and disabilities were included. We searched for these documents at the international and Canadian (federal, provincial, and territorial) levels.

#### 2.1.2. Community-Based Practices

To find community-based practices on inclusive play spaces, we used web-based search (using a combination of the following keywords: disability, children, playground, inclusive, universal accessibility, best practices, recommendations, and guidelines) engines such as Google, to find reports, best practices, guidelines, and recommendations. All different types of functional limitations (i.e., physical, intellectual, communication, and sensory impairments) and disabilities were included. We then reviewed the references listed on the original documents to find further resources.

### 2.2. Surveys, Interviews, and Focus Groups

#### Participants

Participants included school-aged (6–12 years old) children with disabilities and their families, teachers, clinicians, and orderlies (caregiver staff members at school). They were selected from two schools for children with disabilities and one pediatric rehabilitation center in the city of Montreal, Canada. 

A package containing a consent form and two questionnaires (see File S1 for full questionnaire) was sent to all parents and school and rehabilitation staff. The first questionnaire contained closed and open-ended questions relating to perspectives on the current situation of playgrounds in their schools and neighborhoods, the barriers that make playgrounds inaccessible for children, and their recommendations for making playgrounds more accessible. The package sent to parents contained a child-specific questionnaire, through which children were asked about their preferred equipment or types of play activities at playgrounds. The second questionnaire, in the form of a booklet, contained images of four existing inclusive playgrounds with 3–4 elements (e.g., equipment, structure, or feature such as shading area) highlighted in each playground. This booklet provided images of other equipment and elements, such as different types of swings, slides, sensory walls, musical instruments, shades, surfacing, and sand tables. We asked the respondents to choose what they liked and did not like about each playground. We encouraged parents to talk with their children about their ideal inclusive playground and write about it in the booklet. 

In addition, we conducted three focus group sessions with clinicians. We interviewed 10 parents of children with disabilities at playgrounds where their children were present as part of their rehabilitation therapy. The same questionnaire that was sent to participants through the school was used as prompt for the focus group. The open-ended questions were based on a review of the research in inclusive playgrounds [3,11,12,13,14], and they were modified and tailored to the local context by our team members, which included educators, the school principal, and pediatric therapists.

We audio-recorded interviews and focus groups and transcribed them verbatim. We analyzed the quantitative information using IBM SPSS Statistics V23.0 and used NVivo V12.4.0 to analyze the qualitative data. Members of our team read the transcripts independently and generated a coding scheme inductively, on the basis of participants’ responses.

### 2.3. Data Analysis

We developed a data extraction sheet to identify relevant information from the policy documents and community-based practices (see File S2 for full data extraction sheet). We conducted a content analysis [13] with the information extracted to find the current policies and practices related to inclusive playgrounds.

We conducted a descriptive analysis [14] to identify the most salient items related to what makes “dream inclusive playgrounds” free of barriers for children with disabilities and their entourage. 

The first author iteratively analyzed all data points (individual interviews, focus groups, and open-ended responses to questionnaires) and coded the content using NVivo software. The questions asked for the three types of data sources were similar, which allowed a coding structure to be developed. An initial inductive coding structure was created in discussion with the other authors. The remaining data were coded by the first author deductively, in constant consultation with the other authors during regular meetings. Data exported to NVivo were from the three data sources (questionnaires open-ended questions, focus groups, and interviews), and only the information about “role” (e.g., parent, child, or educator) was maintained for confidentiality. The team met regularly to validate iterative results and interpretations, and to provide perspective on the data coding and analysis. Discussions were held with the team to describe how the codes were applicable to all three data sources in order to synthesize and summarize the data.

## 3. Results

### 3.1. National and International Policies

Among the policy documents that we retrieved, two of them were at the international level [7,15], one was at the federal level [16], and seven were at the provincial level [17,18,19,20,21,22,23]. Both international policies were conventions developed by the United Nations General Assembly, namely, the Convention on the Rights of the Child and the Convention on the Rights of Persons with Disabilities [7,15]. In both conventions, numerous articles address the rights of children with disabilities. Specifically, Article 31 in the UN CRC and Article 30 in the UN CRPD explicitly emphasize their rights to play, participate in leisure activities, and enjoy recreational opportunities. As of July 2023, 140 countries signed and 170 countries ratified the UN CRC. The total numbers of the countries that have signed and ratified the UN CRPD are 164 and 186, respectively [24]. Typically, the signing of these conventions serves as the initial stage of accepting the international act or treaty, and the subsequent process of ratification indicates the signatory agreement of the state to be legally bound by the treaty upon its entry into force.

At the federal level, the Canadian Standards Association developed a National Standard of Canada for children’s play spaces and equipment. This standard includes an Annex H that provides minimum accessibility requirements for children with disabilities in public play spaces, although they are not mandatory [16]. Annex H is most suitable to be applied for the development of new play spaces, but it can also be utilized for renovation or retrofit projects. The first part outlines accessibility requirements for children with disabilities, encompassing both ground and aboveground areas of the play space. In the second part, the Annex provides detailed specifications on how to fulfill these accessibility standards. 

At the provincial level, only four provinces had developed policies regarding accessible or inclusive play spaces. New Brunswick put forward a wellness strategy in 2014 which included initiatives, programs, and services working to increase and encourage physical activity and participation in sport and recreation for all children [20]. Nova Scotia passed an accessibility act in 2017, which includes policies on play spaces in the built environment [18]. Policies related to the built environment encompass the human-made spaces where people live, work, learn, and play. The objective of the Accessibility Act is to ensure that Nova Scotia becomes an inclusive and barrier-free province by the year 2030. This involves the development of six accessibility standards, which are currently under development. Ontario has a regulation on the construction of accessible play spaces, known as the Integrated Accessibility Standards, developed under Accessibility for Ontarians with Disabilities Act, 2005. This regulation includes accessibility standards for six areas of information and communications, employment, transportation, the design of public spaces, and customer service. The document has specific considerations regarding outdoor play spaces, consultation requirements, and accessibility features. This document is also accompanied by a regulation guideline that provides further details on how to follow the regulation [17,21]. Quebec has two policies, put forward in 2009 and 2016, “Equals in Every Respect: Because Rights Are Meant to Be Exercised” and “Quebecers on the Move: Policy on Physical Activity, Sport, and Recreation”, respectively, which both emphasize the importance of leisure activities for children with disabilities and contain policies on the rights of persons with disabilities to recreation and leisure activities [19,22]. In 2019, Quebec further provided practical recommendations on both accessible design of area and equipment or elements to be installed on playgrounds [23].

### 3.2. Community-Based Recommendations

Community-based recommendations included guidebooks and toolkits developed by various play-related organizations. Combined, the five community-based recommendations we found addressed the various phases in the delivery of play spaces for children with different abilities and their families. The retrieved guidebooks and toolkits can be classified into recommendations for physical and social environments. Recommendations for the physical environment addressed the physical layout and landscape of play spaces [25,26], sensory features [25,27], play equipment [25,26,27,28,29], support features of the surrounding environment, and community accessibility [25,26,29]. Recommendations for the social environment included activity design concepts and ideas which promote inclusion and full participation among all children [25,26,28]. Additionally, two of the guidebooks addressed the planning process necessary for building inclusive playground, with recommendations from the creation of a planning committee to the recruitment of consultants (play space designers, children, adults, and caregivers), budgeting guidelines, and selection of a physical site for playground building [25,27].

### 3.3. Stakeholders’ Perspectives

In total, we collected 97 completed questionnaires from stakeholders, including children with disabilities, their parents, clinicians, teachers, and orderlies (caregiver staff members at school) (Table 1). In addition, 36 clinicians participated in three focus group sessions and 10 parents of children with disabilities were individually interviewed at public playgrounds where their children were playing as part of their therapy. The children’s ages ranged from 2 to 17 years old (mean 8.3) with various disabilities. Participant demographics can be viewed in Table 2. 

The factors suggested by participants to make playgrounds more inclusive were categorized into three main areas: physical, social, and political environment. A detailed breakdown of these categories follows. 

#### 3.3.1. Physical Environment

**Equipment:** This category was subdivided into diversity and accessibility of equipment.

**Diversity:** A diverse set of equipment, with varying degrees of difficulties, would allow children with different abilities and their siblings to play together. This diversity would stimulate all senses, including proprioceptive, tactile, visual, vestibular, auditory, fine motor, and gross motor. 

**Accessibility:** It was indicated that most of the equipment in playgrounds is not accessible nor usable for children with different abilities; almost all equipment is off the ground and can only be accessed by ladder or other climbing features. Recommendations included making equipment accessible, e.g., by installing an elevated sandbox or installing metal slides, as plastic slides can affect the hearing aids of children with cochlear implants. Equipment that is not accessible may prevent children from engaging in activities with others: As a parent shared: *“None of the equipment like spinners or swings has sufficient support for kids like mine. They need straps and more lateral support”.* A clinician also mentioned that *“there is no adapted equipment for kids in wheelchairs besides ramps”.*

Table 3 indicates the types of equipment and elements that were suggested by participants as facilitators for inclusive play.

##### Design

The layout of the ground and overall area where public play spaces are built must consider the children, families, and others who will be interacting and playing. Considerations go beyond equipment and should include the accessibility of parking lots, pathways, surfaces, and the experience of play that will happen, i.e., where children have to go to use the facilities, what is the journey like to rest areas, and what are the safety concerns that must be planned in advance. Participants also mentioned considering the sensory overload of many public spaces, and considering that, for children who have sensory processing disorders, the design of the space also influences the types of interactions that are possible in the space. Table 4 outlines the design elements that were brought up by participants as essential in creating play experiences that are inclusive. 

**Moveable equipment:** Participants were also asked about other forms of structures that may be moveable such as loose-part playgrounds. Loose parts refer to any materials that can be moved, carried, or altered. These include sticks, rocks, cardboard boxes, ropes, tires, and hay bundles, all of which are cost-friendly and easy to find. Many participants suggested that it could stimulate creativity and develop imagination and play skills. A clinician explained that *“everyday objects promote imagination, giving the message that fun can be found through simple objects, not just the latest toys”.* However, some participants showed concerns around the safety and hygiene of loose parts, as items can be easily misplaced, stolen, or vandalized if materials are not secured. Everyday supervision of setup, takedown, and cleanup is required. Loose playgrounds would make it hard for children with visual impairments and with mobility aids to navigate around space. It was suggested that loose-part playgrounds target more typical children who can independently interact with the objects and are more challenging for children with disabilities. Therefore, consideration of their accessibility is required.

#### 3.3.2. Social Environment

**Advocacy and awareness:** Advocacy and raising awareness by different means, including social media, were found to be essential to promoting an inclusive society. Having discussions with the community about the benefits of inclusive playgrounds for society, focusing on success stories of children with disabilities being included in their community, creating parents’ advocacy groups, and educating children about different disabilities, were all recommended strategies. 

**Attitude:** Attitudes of other children and parents toward children with disabilities have a huge impact on the feelings of children with disabilities. Many parents do not bring their children to playgrounds because of not only the physical barriers but also social barriers their children face at playgrounds. Children should be educated about different types of disabilities and inclusive communities.

**Social play:** Collaborative teaming involves two or more children playing together with a common goal. Participants noted the importance of children being able to play together, and that the environment and equipment in playgrounds should promote and encourage cooperative play and socialization. 

**Communication**: The ability to interact and play with other children, explain rules and objectives, or solve problems depends on an environment that affords easy communication among children. One of the parents explained: “*He [her son] loves to communicate but has a hard time being understood.”* Another parent mentioned that *“she [her daughter] can communicate if she can see them [other kids], because she is deaf”*.

#### 3.3.3. Political Environment

**Getting stakeholder perspectives:** Participants suggested that, when designing playgrounds, city authorities should consult with all stakeholders, including parents, children with disabilities, educators, and clinicians, to listen to multiple perspectives in responding to child’s needs. Parents mentioned the difficulties they face when taking their children out of wheelchairs and lifting or carrying them around playgrounds, causing frustration for their child as they are not able to play or interact with other children. A parent mentioned that her daughter *“just sits and watches the other kids play”.* They shared their hopes that inclusive playgrounds will become the norm and children will not feel isolated: *“I dream that my son can do as many activities as possible.”* (parent).

## 4. Discussion

The policies and regulations identified in this study along with multiple stakeholders’ voices indicate that children with disabilities face limitations in accessing and participating in the physical and social aspects of playgrounds, as well as engaging in activities and play with their peers. Our findings reveal that parents, teachers, and those in the ecological systems of children have a clear idea of barriers that hinder opportunities for inclusion and practical ideas for implementing best practices by tackling the physical environment through accessible equipment and design, the social environment by creating opportunities for interaction and participation of children with different disabilities, and the political environment by consulting with stakeholders and people with lived experiences when creating policies and regulations.

The social model of disability posits that disability is not related to a particular medical condition, but a consequence of societal barriers that disable individuals. Eliminating these “disabling barriers” can open up greater opportunities for persons with disabilities to experience inclusion and full participation in society [30]. The process of inclusion of children with disabilities in public play spaces depends on making the environment fit the child, regardless of their abilities, and creating spaces where all children can have meaningful experiences, regardless of their body function. It is up to every society to find ways of making environments where everyone is welcome and has a sense of belonging [31].

Moore and Lynch also divided the factors influencing inclusive playgrounds into three main areas: physical, social, and political environments [11]. We analyzed participants’ responses in this study in the same categories. Studies demonstrated that accessible playgrounds could provide occupational and social justice for children with disabilities [31,32,33]. However, as related by our study participants and other studies, most playgrounds do not support play activities for children with multiple disabilities. Previous research also shows that, in relation to the political environment, there are insufficient policies and guidelines related to accessible playgrounds, insufficient knowledge of disabilities, lack of funds to design and create accessible playground, insufficient knowledge on how to design inclusive playgrounds, and exclusion of children with disabilities and their families by decision makers. All these constitute barriers to inclusive playgrounds [3,8,9,10,31,32,33,34,35,36,37,38,39,40,41,42,43,44,45,46,47,48,49,50,51]. 

The Community Wellbeing Framework is one example of comprehensive framework conceptualized based on domains related to the social, economic, environmental, cultural, and political conditions required for people and their communities to reach their potential and achieve collective wellbeing [52]. Increasingly, people are acknowledging the influence of built environment design on health and wellbeing, along with its potential long-term effects on quality of life. Our study results highlight the impact that the social domain can have in enabling individuals and their communities to flourish and fulfill their potential. Inclusive playgrounds can be such spaces to promote social growth from childhood and across the lifespan, which emulate social relationships and help children with and without disabilities learn about each other and learn how to create social relationships that value the differences. This attitude can collectively contribute to the social wellbeing of communities [53]. 

In a broader understanding of the environmental domain of the Community Wellbeing Framework, the wellbeing of the environment is linked to the wellbeing of people, and one is not possible without the other. Inclusive playgrounds are places where children can enjoy high-quality experiences, promoting healthy behaviors such as celebration and care for natural and cultural heritage, maximizing physical and visual connections to nature. Connections to nature through play have multiple benefits to children such as impacts on physical activity and cognitive development [54]. Most playground spaces described in our study did not have connections with nature, although this feature was mentioned by some participants as possible facilitators or desired features of inclusive playgrounds. Including opportunities for nature play in playgrounds can help addressing goals such as physical and cognitive development, which children with disabilities frequently only develop in specialized care or rehabilitation, but could be developing organically in their communities with much more intensity and equity [55]. 

Inclusive playgrounds promote a sense of ownership and integration for the disability community. They are also a product of collaborations with decision makers and various professional disciplines, stakeholders, and constituents who have been meaningfully engaged from the early stages of design process.

### 4.1. Children with Disabilities in Design of Physical Environment

Our findings revealed the importance of design in making playgrounds inclusive. Previous studies showed that the design of an inclusive playground is usually an afterthought and that designers do not have enough knowledge about inclusive playgrounds [8,9,11]. Universal design (UD) could be an answer to the design of such inclusive play spaces, a model advocating “the design of products and environments to be usable by all people, to the greatest extent possible, without the need for adaptation or specialized design” [56]. In UD, the goal is to acknowledge human diversity and find solutions to ensure that environments and objects are accessible and usable for all individuals. [31]. Designers should give attention to accessibility from the onset of the design process and include feedback from children, families, and other stakeholders. 

### 4.2. Importance of Play and Elimination of Social Barriers 

Through play, children can practice social roles, communication, leadership, and obedience, allowing them to learn the rules of social relationships and community building. Therefore, children with disabilities should be afforded inclusive playgrounds to enjoy their rights to play and to obtain positive impacts in their lives. Our study, along with previous research, found that social environments could be barriers to providing such play spaces. As awareness, advocacy, and attitude were found to be essential factors, it is important to empower children and their families and other stakeholders to advocate for inclusive playgrounds, and to reach out to policymakers, their local government, and municipalities to make playgrounds accessible. Furthermore, it is necessary to educate communities about people with disabilities and their needs to establish a positive attitude toward creating and promoting an inclusive society.

### 4.3. The Lack of Policies on Play

Children’s right to play is officially protected by the UN CRC and UN CRPD. As a signatory country, Canada must act and report on advances in implementing those conventions. Bill C-81, also known as the Accessible Canada Act, was approved in June 2019 and constitutes a positive step forward from the federal government toward improving the lives of Canadians with disabilities [57]. However, children with disabilities and their right to play are not mentioned in this act. While the act promotes positive change by ensuring accessibility in public buildings, it does not currently extend to playgrounds, leaving room for improvement in this area.

Previous studies showed that European, British, and Australian playground standards lack guidance on ensuring accessibility for disabled children, in terms of both playgrounds and the equipment used [58]. In Canada, the only available accessibility guideline for playgrounds is provided by the Canadian Standards Association, through Annex H. Annex H was developed closely on the basis of the Americans with Disabilities Act (ADA): Accessibility Guidelines for Play Areas, in terms of both content and layout [16]. However, as opposed to ADA, Annex H is not mandatory. Moreover, it essentially demonstrates how to make playgrounds accessible for children with a mobility restriction, with limited options for the needs of children who have other disabilities. While all provinces have specific policies related to disabilities, very few have addressed items such as leisure activities or play within their policies. As argued by Shikako-Thomas and Law [44], the emphases of disability policies in Canada are mainly “adult-based”, mostly concerning employment, health services, independent living, and transportation. 

### 4.4. Opportunities to Play

Many participants in this study suggested that a variety of activities and play opportunities with some degree of risk or challenges should be provided at inclusive playgrounds. However, many providers of playgrounds are often more concerned about the safety and legal consequences which might result from an accident [48,59], than about the value of play. As clinician in our study suggested: *“When thinking about an accessible playground, it doesn’t mean it has to be boring.”* Our goal should, therefore, be making playgrounds inclusive so all children can have fun, have opportunities to play with peers, develop their imagination, socialize, feel a sense of belonging, and, most importantly, do what children are supposed to do: play! 

## 5. Conclusions

On the basis of the findings of this study, we concluded that, although play is crucial, and although children with disabilities have the right to inclusive playgrounds, there is a lack of sufficient policies and guidelines mandating such provision. This prevents children with disabilities from playing and interacting with their peers. There is a need to empower children and youth with disabilities and their families through rights-based education to advocate for themselves effectively. In addition, there is a need to train educators, healthcare providers, and policymakers on the social model of disabilities and the notion of the rights of children to play. All stakeholders, including children with disabilities and their families, researchers, healthcare workers, educators, policymakers, designers, and leisure practitioners, should be involved in decision making about inclusive playgrounds. Action should be taken to develop, implement, and regularly monitor the efficacy of policies and guidelines for inclusive playgrounds to ensure they facilitate the active participation of children with disabilities in play. Provision of inclusive playground environments, including addressing physical, social, and political factors, should be a priority to maximize participation of children with disabilities in societies and to enable them to enjoy their rights to play.

## 6. Limitations and Future Directions

Getting feedback from children as part of a research project, either formally or informally, is an extremely difficult procedure; although the parents were informed about the project and its benefits for their children in advance, the response rate from parents and children was only 23%. Another limitation was ecological validity, which is the extent to which the findings of a research study can be generalized to other settings [60]. However, this study did not aim to establish generalizability but rather obtain an in-depth account of participants in this setting to gain perspectives on current situation of playgrounds, as well as barriers and facilitators for inclusive playgrounds. It also informed immediate needs to rebuild play spaces, providing both an evidence-based review of research and regulations and a thorough account of stakeholder-identified needs. Future directions might include understanding how policies play a vital role and developing specific inclusionary strategies for playgrounds at municipal and national levels. Further study of policy issues might also help to encourage awareness at the political and economic level.

## Figures and Tables

**Table 1:** Gender and total number of participants.

Participants	Total (*n*)	Sex (Female, *n*)
Parents and children	36	24 (parents) 16 (children)
Clinicians	36	36
Teachers	18	14
Orderlies	7	6

**Table 2:** Types of disabilities of the children * (*n* = 36).

Types of Disabilities	%
Motor	26
Communication	26
Learning	11
Intellectual	9
Auditory	9
Behavioral	8
Visual	5
Autism spectrum disorder	3
Others	3

* Some children had more than one type of disability.

**Table 3:** Types of equipment or elements suggested by participants.

Suggested Equipment/Elements	Special Considerations/Quotes
Bench and rest area	Providing a quiet place or zone for kids who do not want to play or need to rest. More seating areas and picnic tables should be provided for parents. *“There are no benches for parents or caregivers to sit on. My child doesn’t like it if I am standing right over him.”* (parent).
Drinking fountain	Must be wheelchair-accessible.
Washroom	Wheelchair-accessible and be provided with adult changing tables for older/bigger children in addition to baby changing tables, which should also be wheelchair accessible for parents with disabilities.
Shade	Many children with visual impairments have a sensitivity to sunlight, and some other children cannot tolerate heat. Implementing shades would also allow playgrounds to be more usable in various weather conditions. Both play and rest areas should be covered, protecting children, parents, and educators from the sun when playing, eating, talking, and resting, to help them relax and avoid heatstroke. *“It is important to have proper shades that can be moved around according to the sun and children’s position.”* (clinician).
Handgrip	Handgrips and handrails should be present for ramps and various equipment.

**Table 4:** Design elements suggested by participants.

Suggested Design Elements	Special Considerations/Quotes
Layout and pathway	There should be a large, flat open space for children, so they have better mobility to run and move wheelchairs around the playground. Asimple, visually calming, and nonoverwhelming layout would enable parents to keep track of their children.
Surface	A vast majority of participants declared accessible surfaces to be one of the main facilitating factors for inclusive playgrounds. They recommended replacing woodchips, grass, and sand with accessible surfaces such as a poured-in-place rubber surface and to remove steps and make the surface even and padded, such that all play space sections are connected, including the resting area and parking. Ground markers with contrast and texture would enable visually impaired children to trail and navigate space. Providing a tactile or visual boundary around unsafe elements such as swings was also suggested. *“Markers on the ground to indicate a raised walkway or bridge. Otherwise, people who are blind are more likely to walk into them.”* (teacher).
Ramp	Providing large accessible walkways and ramps with gradual and non-steep slopes instead of stairs that connect all elements was recommended. Providing separate entrance and exit ramps at structures and putting exit ramps near the slides would avoid backtrack for parents, creating less traffic and confusion around structures. *“A ramp near the slide that parents would come down with the child at the same time and also something that keeps the child safe until the parent reaches them.”* (parent).
Color contrast and lighting	High color contrasts and lighting for pathways and equipment perimeters improve visual accessibility and make it easier to maneuver around the playground with mobility aids. The color contrast was suggested for handgrips and on certain pieces of equipment. However, it should be noted that too many colors can be misleading for children with sensory impairments.
Safety	Safety was a concern for many participants; being able to see the child is important for both the child and parents and is facilitated in an uncluttered playground. Having a fence with one entrance and exit for playgrounds was suggested. *“Usually parents are supervising the kids, but if the parent is with several kids, it is better to have a fence, especially for the smaller kids; they don’t know the clear boundaries.”* (clinician). Enough personnel to monitor and guide children, providing frequent equipment maintenance, fixing broken equipment, sharp edges, bumps, or holes in the ground, and taking action on the presence of bees, bugs, and poison plants were recommended.
Overstimulation	Too much light, flashing, or rapid movement is a trigger for children with seizures. Additionally, too much noise may cause discomfort to children with autism spectrum disorders.
Proximity to home	The proximity of the playground to home is an important factor to allow access and actual use of the playground.
Parking	Providing parking space with accessible pathways to play space can ease navigation.
Incorporating nature	Nature-inspired playgrounds are preferred to industrial metal framed ones; trees create natural shade, bringing birds and a sense of peacefulness. Such environments are also aesthetically pleasing and alter children’s energy in positive ways. Participants suggested adding rocks, wood elements, more trees, and grass. “*It would be nice for kids to be able to touch trees and rocks [as well as] natural pieces integrated into play areas. It looks too artificial otherwise.”* (clinician).
Instruction and guidance	The importance of training children on how to use certain equipment was noted. Instructions could be provided by staff, as well as different types of animations or videos. The instructions could also be provided on boards or QR codes installed close to equipment. During the focus group sessions, the clinicians discussed the importance of bringing children with disabilities to playgrounds with therapists, introducing different equipment and play opportunities, showing them boundaries, and encouraging children to interact with their peers, to support them while at playgrounds. These therapy sessions at playgrounds not only help the children to overcome difficulties while playing but also increase the confidence of both children and their families.

## Data Availability

Data sharing is not applicable to this article.

## Supplementary Materials

The following supporting information can be downloaded at: https://www.mdpi.com/article/10.3390/children10081308/s1, File S1: List of questions; File S2: Summary of Policies and Community best practices
