# Supplementary material for: Promoting Health through Accessible Public Playgrounds
Source: Children (Basel). 2023 Jul 29;10(8):1308. doi: 10.3390/children10081308 (PMC10453442; doi:10.3390/children10081308)
Supplement: Supplementary file 1 [file children-10-01308-s001.zip › children-2510475-supplementary/File S1_List of questions.pdf]

Questionnaire for children with disabilities

1. City
2. Borough (District)
3. Age
4. Gender ☐F ☐M ☐Other
5. Types of impairments/issues

☐ Intellectual
☐ Motor
☐ Visual
☐ Behavioral
☐ Autism Spectrum Disorder
☐ Learning
☐ Communication
☐ Chronic Diseases
☐ Other
Specify: _____________________________

6. Registered in a special needs school or program ☐Yes ☐No ☐N/A
School name:

Thinking about the playground you use the most

1. What kind of equipment do you prefer at the playground? Check all that apply.
☐ Slide
☐ See Saw
☐ Springer
☐ Roundabout/ Spinner
☐ Swing
☐ Sand pit
☐ Others
Specify: ___________________________

2. Please list the top 3 things that help you to play in playgrounds (parking, light, shade, drinking fountain, ground surface, pathway, elements, colors, etc.)?
3. Please list the top 3 things that prevent you from playing in playgrounds (parking, light, shade, drinking fountain, ground surface, pathway, elements, colors, etc.)?
4. Do you find it easy to play on your preferred equipment?
☐ Yes ☐ No
Why?
5. How often do you go to playground?
☐ every day
☐ once or twice a week
☐ once or twice a month
☐ every few months
☐ never
6. How do you get to the playground?
☐ I usually go by car with my parents

☐ I usually go by car with someone else
☐ I walk/wheel to the park with my parents
☐ I walk/wheel to the park with my friends or someone else
☐ I only go to the playground at school

7. How much do you enjoy playing in your neighborhood playground?

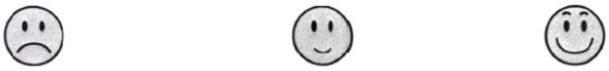

☐ Not at all ☐ Neutral ☐ a lot

8. Are there any sound play or musical instruments at the playground where you play the most?
9. Is there any equipment you wish you could use but usually can't use? (Briefly explain)
10. What would you suggest to make the playground more accessible and usable for children with motor, visual, hearing, communication, intellectual impairments?
11. Is it easy for you to play with other children at the playground?

☐ Yes ☐ No ☐ I don't know

Why?

12. Is your neighborhood playground accessible for children with wheelchair or with a walking aid?

☐ Yes ☐ No ☐ I don't know

Why?

13. Is your neighborhood playground accessible for children with visual impairments?

☐ Yes ☐ No ☐ I don't know

Why?

14. Do you usually feel safe at the playground?

☐ Yes ☐ No ☐ I don't know

Why?

15. Any other comments about playgrounds and public play spaces?
16. Do you have a particular story (positive or negative) or example of how a public play space influenced you to play and have fun?

Questionnaire for parents/caregivers of children and youth with disabilities

Questions 1-4 are about your child/children with disabilities

1. Age
2. Gender ☐F ☐M ☐Other
3. Types of impairments/issues
   ☐ Intellectual
   ☐ Motor
   ☐ Visual
   ☐ Behavioral
   ☐ Autism Spectrum Disorder
   ☐ Learning
   ☐ Communication
   ☐ Chronic Diseases
   ☐ Other
   Specify: ______________________________
4. Registered in a special needs school or program ☐Yes ☐No ☐N/A

School name:

Parent/ Caregiver

1. City
2. Borough (District)
3. Age

☐Under 25

☐25-29 years old

☐30-34 years old

☐35-39 years old

☐40-44 years old

☐45-49 years old

☐50 years old and up

4. Gender ☐F ☐M ☐Other ________________

5. Highest level of education (of parent/caregiver)

☐High School Diploma

☐Post-Secondary/ Professional Degree (Not University)

☐University Degree

☐Post-Graduate Degree

For all questions, please consider the playground or public play space (e.g. parks, or anywhere kids usual play - in your neighborhood or school) that your child uses the most.

1. How often does your child play in a public play space such as neighborhood playground, water park, public park? (Other than school)

☐ every day

☐ once or twice a week

☐ once or twice a month

☐ every few months

☐ never

2. What kind of equipment does your child prefer to use in playgrounds or play spaces (e.g. public playgrounds, play spaces and parks in your neighborhood, or in school)? Check all that apply.

☐ Slide

☐ See Saw

☐ Springer

☐ Roundabout/ Spinner

☐ Swing

☐ Sand pit

☐ Others _____________________

Why?

3. Please list the top 3 features that help your child to play in playgrounds (Parking, light, shade, drinking fountain, ground surface, pathway, elements, colors, etc)?

4. Please list the top 3 features that prevent your child from playing in playgrounds (parking, light, shade, drinking fountain, ground surface, pathway, elements, colors, etc)?

5. What equipment do you wish that your child could use, but is not accessible to him/her? Why?
6. Thinking of your child's needs, what might you suggest would make playgrounds more accessible and usable?
7. Thinking of other children with motor, visual, hearing, communication, and intellectual disabilities, what might you suggest would make the playground more accessible and usable?
8. Is it easy for your child to interact with peers in playgrounds?

☐ Yes ☐ No

Why?
9. Is your neighborhood playground and/or school playground equipment and space accessible for children who use a wheelchair or walking aid?

☐Yes ☐ No ☐ I don't know

What makes it accessible or not?
10. Is your neighborhood playground and/or school playground equipment and space accessible for children with visual impairments?

☐Yes ☐ No ☐ I don't know

What makes it accessible or not?
11. Do you think your neighborhood and/or school playground equipment and space are safe for your child?

☐ Yes ☐ No ☐ I don't know

Why?
12. Do you believe that the playground environment in general:
a. creates positive/negative feelings for your child?

☐Positive

☐Negative

☐Neutral

Why?
b. promotes/limits development and free play for your child?

☐ Promotes

☐ Limits

☐ Neutral

Why?
13. Any other comments about playgrounds and public play spaces?
14. Do you have a particular story (positive or negative) or example of how a public play space influenced your child's ability to play and have fun?

Questionnaire for clinicians/educators/orderlies

1. City
2. Borough (District)
3. Age

☐Under 25

☐25-29 years old

☐30-34 years old

☐35-39 years old

☐40-44 years old

☐45-49 years old
☐50 years old and up
4. Gender ☐F ☐M ☐Other
5. Highest level of education
☐High School Diploma
☐Post-Secondary/ Professional Degree (Not University)
☐University Degree
☐Post-Graduate Degree

Thinking of the school playground

1. Do you believe that the current playground is accessible and usable for children with different abilities?

☐ Yes ☐ No ☐ I don't know
What makes it accessible or not?

2. Do you think the playground area is safe for children with different abilities?

☐ Yes ☐ No ☐ I don't know
Why?
3. In your opinion, which social barriers prevent children with different abilities to play in public spaces?
4. Thinking of children with motor, visual, hearing, communication, and intellectual impairments, what would you suggest to create an ideal playground?
5. What strategies would you suggest to advocate for accessible playgrounds?
6. Do you believe that the playground environment in general:
a. creates positive/negative feelings for children with disabilities?
☐Positive
☐Negative
☐Neutral
Why?
b. promotes/limits development and free play for children with disabilities?
☐ Promotes
☐ Limits
☐ Neutral
Why?
7. Have you considered bringing, or have you brought, children to public playgrounds as part of their therapy? (question not for educators or orderlies)
8. Have you served as a consultant for municipalities or private playground designers on practical issues of building inclusive playgrounds? (question not for educators or orderlies)
9. Any other comments about playgrounds and public play spaces?
