# Supplementary material for: Promoting Health through Accessible Public Playgrounds
Source: Children (Basel). 2023 Jul 29;10(8):1308. doi: 10.3390/children10081308 (PMC10453442; doi:10.3390/children10081308)
Supplement: Supplementary file 1 [file children-10-01308-s001.zip › children-2510475-supplementary/File S2_Summary of Policies and Community best practices.pdf]

Summary of data extraction

Table S1. Summary of policies

Level	Province	Name	Year	Type	Summary
**International**		United Nations General Assembly: Convention on the Rights of the Child	1989	Convention	*Contains articles pertaining to the rights of children with disabilities to play
		United Nations General Assembly: Convention on the Rights of Persons with Disabilities and its Optional Protocol	2006	Convention	*Contains articles pertaining to the rights of children with disabilities to play
**Federal**		Children's playspaces and equipment standard. CAN/CSA-Z614-14. National Standard of Canada	2015	Standard	*Annex H provided minimum accessibility guidelines for newly constructed playspaces and renovations and retrofits to existing playgrounds.
**Provincial**	**NB**	New Brunswick's Wellness Strategy 2014-2021	2014	Strategy	Includes initiatives, programs and services which work to increase and encourage physical activity and participation in sport and recreation.
	**Nova Scotia**	Accessibility Act. Bill No. 59. An Act Respecting Accessibility in Nova Scotia.	2017	Act	*Policies pertaining to the 'built environment' include human-made space in which people live, work, learn and play and includes buildings, rights-of-way and outdoor spaces.
	**Ontario**	Ontario Regulation 191/11: Integrated Accessibility Standards based on the Accessibility for Ontarians with Disabilities Act.	2005	Regulation	*Provides regulations for constructing accessible play spaces in Ontario.
		A Guide to the Integrated Accessibility Standards Regulation.	2014	Regulation guideline	*Provides overview of regulations in Ontario for constructing accessible play spaces.
	**Quebec**	Equals in Every Respect: Because Rights Are Meant to Be Exercised	2009	Policy	Contains policies on the rights of persons with disabilities to recreation and leisure activities

		Quebecres on the Move; policy on Physical Activity, sport and Recreation	2016	Policy	Contains orientation to facilitate participation of people with disabilities in recreation and leisure activities
		Guide d’accompagnement Mobilier Urbain, L’approvisionnement en Biens et Services Accessibles Aux Personnes Handicapées.	2019	Guideline	Provides practical recommendations on inclusive playgrounds.

Summary of Community best practices

Name	Year	Type	Summary
Playworld - Inclusive Play Design Guide (USA)	2015	Guidebook	Guidebook states all guidelines and laws in the World concerning universal play.<br>All sections are relevant for children with ASD, visual & auditory impairments, physical disabilities and others.<br><br>- Section on planning talks about everything from how to create a planning committee to how to recruit expert consultants and how to select a site for playground building.<br>- Section on layout talks about entry/orientation, wayfinding, perimeter containment, orientation path, pods/rooms/zones, color as safety and wayfinding tool, gathering spaces, line of sight, landscaping.<br>- Section on access talks about unitary surfacing, reach ranges, transfer platforms, transfer steps, width of route, flush transitions (surfacing) – mostly for kids with wheelchairs<br>- Section on play richness mentions different activities/ways of playing.<br>- Section on selecting playground equipment discusses elevated play, contiguous/non-contiguous play, resting point near slide, color of play equipment, rich play experiences at all heights, multiple levels of challenge, making the 'coolest equipment' in the playground accessible to all<br>- Section on support features discuss the support systems & built

			environment surrounding the play space that can maximize inclusion (seating/restrooms/fountains/picnic tables/parking/trashcans/shade/cooling devices/service animals/emergencies/alt. transport/signage visibility

Me2 - 7 Principles of Inclusive Playground Design (USA)	2016	Guidebook	Guidebook containing seven principles of Universal Design, originally created by the Center for Universal Design at North Carolina State University, have been uniquely tailored to what all children want to feel and experience during outdoor play. Comprehensive/in-depth guidebook on playground design.<br>7 principles:<br>1. Be fair: The play equipment provides social justice by being equitable and usable by people of all abilities so they can enjoy their right to play.<br>2. Be included: The play environment supports the participation of individuals with diverse abilities in social & physical activities for inclusive, intergenerational play.<br>3. Be smart: The play environment is easy to understand, allowing individuals to be successful and gain confidence through play.<br>4. Be independent: The play environment allows children to effectively explore and participate in play at their own level.<br>5. Be safe: The play environment addresses current safety standards while providing developmental opportunities needed for exploration and challenge.<br>6. Be active: The play environment supports various degrees of physical and social participation in play while minimizing unnecessary fatigue.<br>7. Be comfortable: The play environment is usable for individuals with sensory needs, diverse body size, posture, mobility, and motor control.<br><br>Guidebook includes case studies of successful inclusive playgrounds.
Rick Hansen - Let's Play Toolkit (CANADA)	NA	Guidebook & Toolkit	Comprehensive guidebook for designing universally accessible playgrounds, includes a toolkit that designers can use to make sure their playground is inclusive. Guide also includes a section on the planning and budgeting necessary to construct inclusive playgrounds. Talks about consultation with play space designers, and workshopping (way to consult) children, adults and caregivers. Guide includes a section on case studies of successful inclusive playgrounds.<br><br>Although there is a focus on children with physical disabilities (mostly wheelchair access), attention is given to promoting sensory and

			perceptual motor development by including different textures, shapes, sounds and scents to the playground design.
The Illustrated Technical Guide to the Accessibility Standard for the design of public Spaces. Global Alliance on Accessible Technologies and Environments (GAATES)	2014	Guideline	Provides toolkits, guidelines, regulation overview for design of accessible play spaces.
Landscape Structure - A Higher Level of Inclusive Play (USA)	2016	Inclusive Play Catalog	Playground catalog that includes playground structures and materials that are tailored to children of all abilities.
